# Avoiding pericardiectomy: a case report of successful medical management for tuberculous constrictive pericarditis in the peripartum period

**DOI:** 10.1093/ehjcr/ytag794

**Published:** 2026-10-26

**Authors:** Jose Gabriel Solis, Cecilia Zarazua Sosa, Cristina Valdez Lopez, Oziel Ivan Godoy Garcia

**Affiliations:** Department of Cardiology, Hospital General de Matamoros, Av. Marte R. Gómez, Col. Leyes de Colonos, 87395 Matamoros, Tamaulipas, Mexico; Department of Clinical Research, Instituto de Ciencias y Estudios Superiores de Tamaulipas, Facultad de Medicina, Calle Oaxaca No. 1, Fraccionamiento Victoria, Sección Fiesta, 87390 Matamoros, Tamaulipas, Mexico; Intensive Care Unit, Hospital General de Matamoros, Av. Marte R. Gómez, Col. Leyes de Colonos, 87395 Matamoros, Tamaulipas, Mexico; Department of Obstetrics and Gynecology, Hospital General de Matamoros, Av. Marte R. Gómez, Col. Leyes de Colonos, 87395 Matamoros, Tamaulipas, Mexico; Department of Internal Medicine, Hospital General de Matamoros, Av. Marte R. Gómez, Col. Leyes de Colonos, 87395 Matamoros, Tamaulipas, Mexico

**Keywords:** Pericarditis, Tuberculous, Pericarditis, Constrictive, Postpartum period, Echocardiography, Antitubercular agents, Case report, Tuberculosis

## Abstract

**Background:**

Tuberculous pericarditis (TBP) is a rare but lethal peripartum complication, often masked by physiological gestational changes. Transient constrictive pericarditis represents a reversible inflammatory phenotype that can resolve with medical therapy, potentially avoiding high-risk surgical intervention.

**Case summary:**

A 22-year-old postpartum female presented with acute respiratory distress and was diagnosed with miliary tuberculosis. Echocardiography confirmed classic constrictive physiology. The patient was diagnosed with a transient constrictive phenotype and managed with a standard four-drug antituberculous regimen, oral prednisone (1 mg/kg), and diuretics. Following intensive medical treatment, she achieved complete clinical and haemodynamic resolution, avoiding the need for pericardiectomy. At 6-month follow-up, she remained asymptomatic with normalized echocardiographic parameters.

**Discussion:**

This case highlights the importance of distinguishing the transient inflammatory phase of TBP from chronic constriction in the peripartum period. Using echocardiographic criteria and initiating prompt, targeted pharmacological therapy can lead to successful reverse remodelling and favourable maternal outcomes.

Learning points

- Tuberculous pericarditis can manifest as a transient, reversible constrictive phenotype during the puerperium.
- Echocardiography confirms constrictive physiology, while clinical context and active inflammatory markers suggest potential reversibility.
- Early medical therapy with antituberculous drugs and corticosteroids can achieve complete haemodynamic resolution, avoiding high-risk pericardiectomy.

## Introduction

Tuberculosis (TB) remains a leading global cause of infectious death. Pericardial involvement is particularly lethal, with mortality reaching 40% despite treatment.^1^ The clinical spectrum encompasses pericardial effusion, effusive-constrictive, and chronic constrictive pericarditis. Notably, a distinct phenotype called transient constrictive pericarditis presents with reversible clinical and echocardiographic signs that resolve with medical therapy, obviating the need for surgical pericardiectomy.^2^

While TB is prevalent among women of childbearing age, peripartum constrictive pericarditis is rare, resulting in limited clinical data.^3^ Herein, we report a peripartum case of miliary TB presenting with transient constrictive pericarditis that achieved complete resolution with medical therapy alone, highlighting the importance of prompt diagnosis and medical management in achieving favourable maternal outcomes and avoiding high-risk surgery.

## Summary figure

**Figure:**
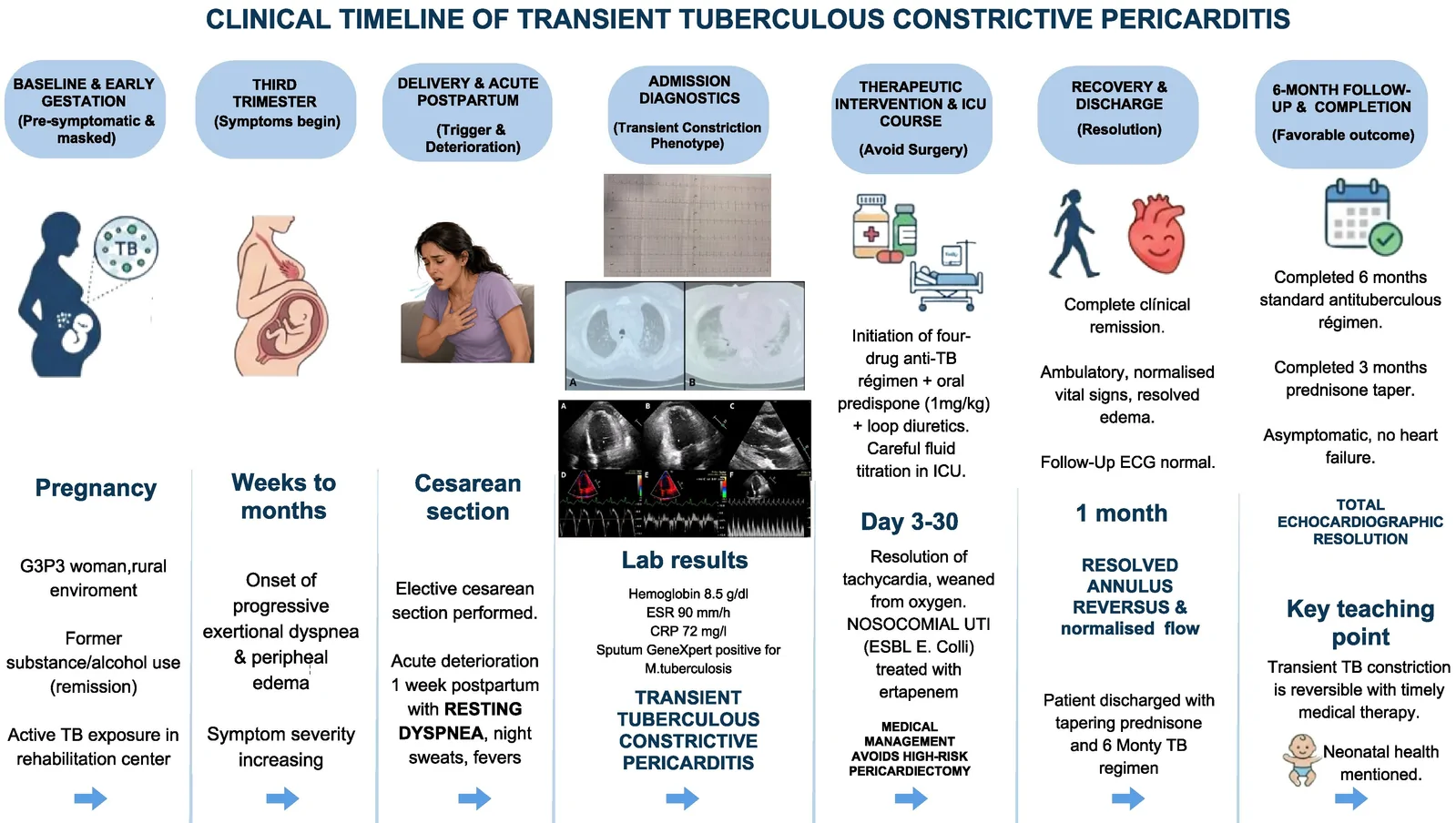


## Case presentation

A 22-year-old G3P3 female from a rural environment presented with acute respiratory distress, 1 week after an elective caesarean section at 38 weeks of gestation. Her history was significant for former polysubstance use (cocaine, marijuana, and solvents) and chronic alcoholism in remission, with recent TB exposure during inpatient rehabilitation. Symptoms began at 34 weeks of gestation with progressive exertional dyspnoea and peripheral oedema, deteriorating acutely 1 week postpartum with dyspnoea at rest, night sweats, and fever. On admission, she required supplemental oxygen (SpO_2_ 80% on room air) and was tachycardic (130 b.p.m.) and tachypnoeic (33 breaths/min) with a blood pressure of 100/60 mmHg. Physical examination revealed prominent jugular venous distension, a positive Kussmaul sign, muffled heart sounds, and 4+ pitting oedema.

Laboratory evaluation showed microcytic anaemia (haemoglobin 8.5 g/dl, reference range 12–15.5 g/dl), elevated C-reactive protein (72 mg/l, reference range <5 mg/l), and erythrocyte sedimentation rate (90 mm/h, reference range 0–20 mm/h), with hypoalbuminaemia (3.2 g/dl, reference range 3.5–5 g/dl). Evaluation for underlying immunodeficiency or systemic autoimmunity was unremarkable, with negative serologies for HIV, hepatitis B, and hepatitis C, alongside non-reactive antinuclear antibodies and rheumatoid factor. Electrocardiography (*Figure 1*) demonstrated sinus tachycardia, low-voltage complexes, electrical alternans, and generalized T-wave inversions. Chest radiography and high-resolution computed tomography (CT) confirmed cardiomegaly, bilateral pleural effusions, and a diffuse miliary pattern. CT mediastinal windows demonstrated diffuse pericardial thickening and accompanying effusion without evidence of calcification (*Figure 2*). Thoracentesis revealed a lymphocyte-predominant exudate with an adenosine deaminase (ADA) level of 50 U/l (reference range <40 UI/l). Although pleural fluid acid-fast bacilli smear and molecular testing were negative—consistent with the paucibacillary nature of tuberculous serous effusions—sputum GeneXpert confirmed *Mycobacterium tuberculosis*. Ultimately, the high pre-test probability (classic miliary pattern on CT and close TB contact), combined with the elevated ADA exudate and microbiologically confirmed pulmonary TB, established the diagnosis of pericardial TB in accordance with international guidelines.

**Figure 1:**
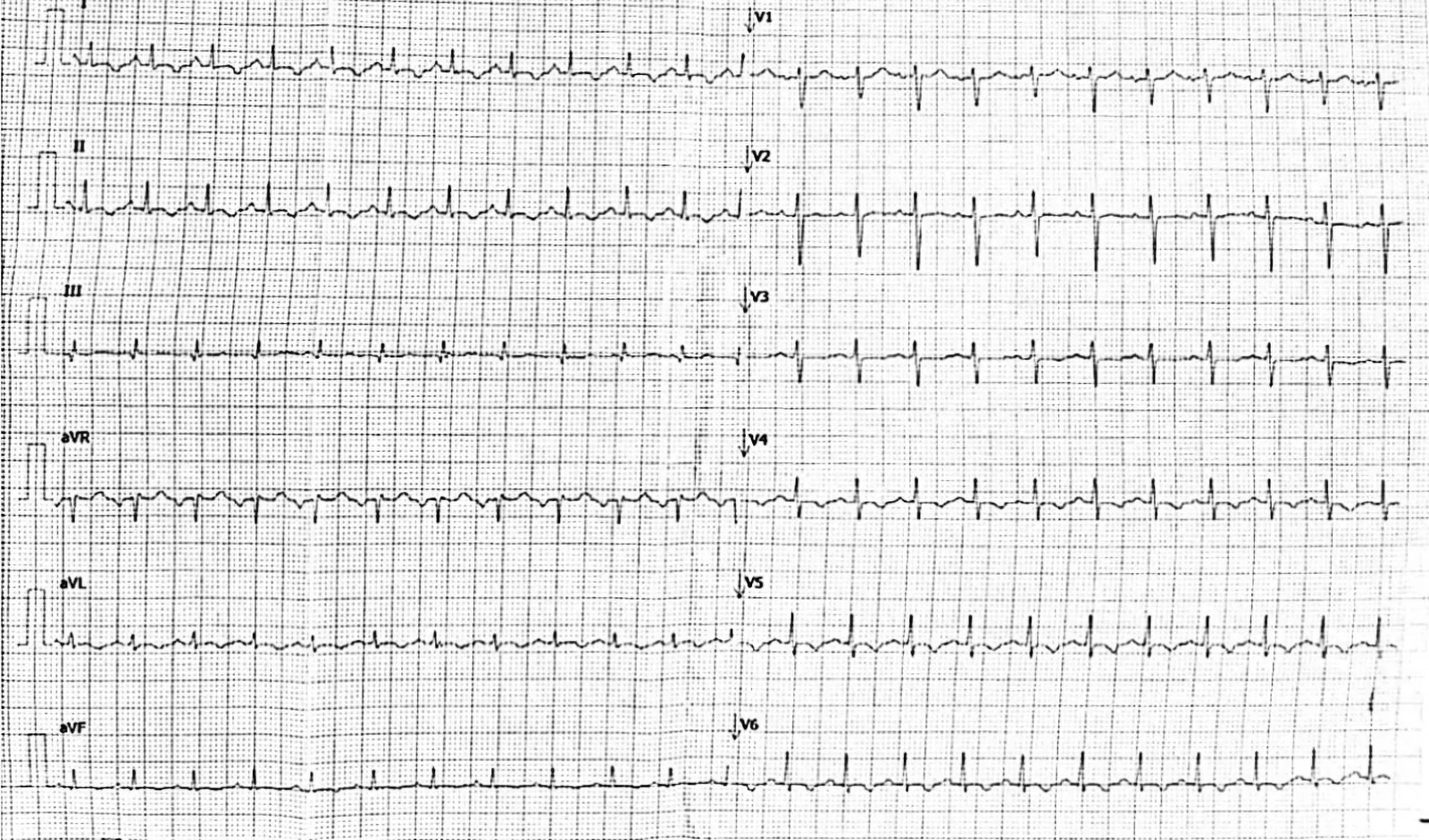
Admission 12-lead electrocardiogram (standard calibration) showing sinus tachycardia at 130 b.p.m. Key electrocardiographic features include: (i) low QRS voltage in the limb leads, (ii) electrical alternans visible across the precordial leads, and (iii) widespread non-specific T-wave inversions, consistent with extensive pericardial inflammation and circumferential effusion.

**Figure 2:**
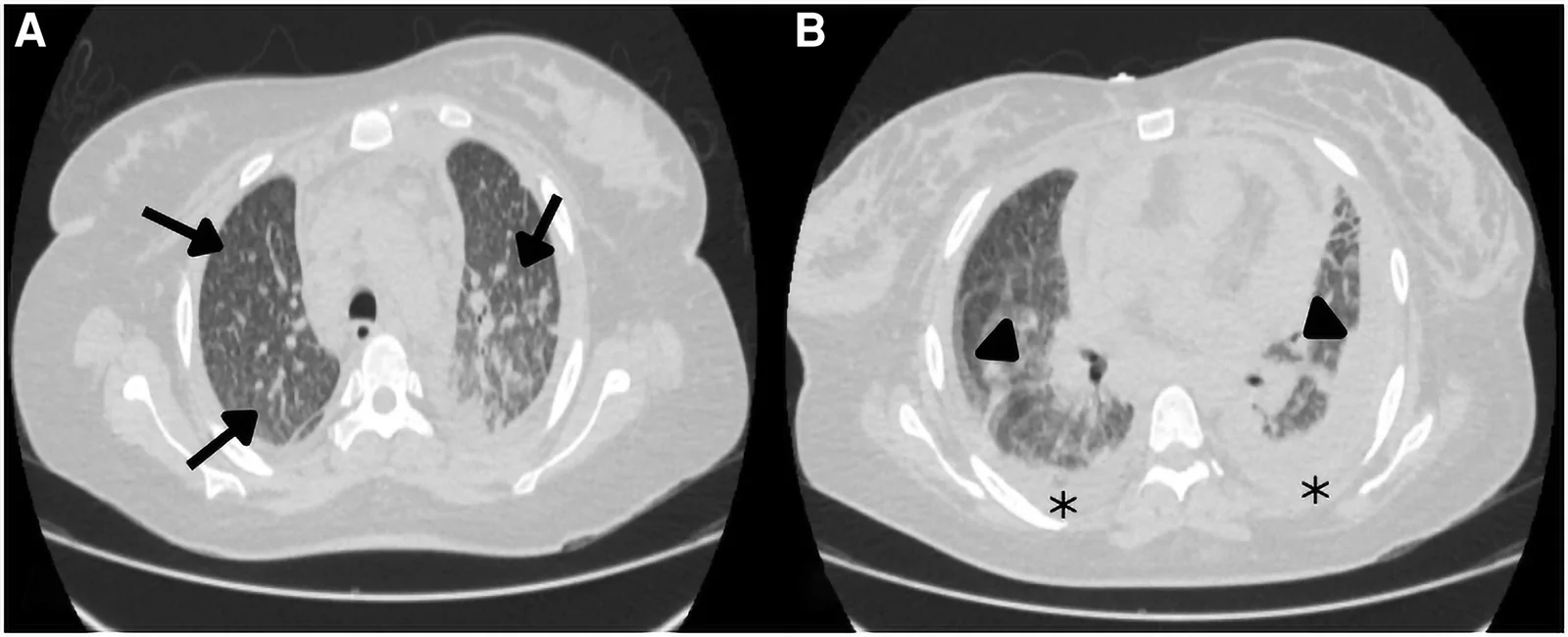
High-resolution axial chest computed tomography (lung window). (*Panel A*) demonstrates a classic miliary pattern with widespread, uniformly distributed, fine micronodules (arrows). (*Panel B*) displays bilateral dependent alveolar consolidations (arrowheads) accompanied by bilateral pleural effusions (asterisks).

Transthoracic echocardiography (*Figure 3*) showed a thickened (7 mm), hyperechogenic pericardium and a 5 mm loculated effusion. Haemodynamic assessment confirmed a constrictive physiology, with a prominent septal bounce, *annulus reversus* (septal e′ 17 cm/s vs. lateral 10 cm/s), significant respiratory inflow variation (mitral 29%, tricuspid 51%), and expiratory hepatic vein flow reversal. Given the subacute symptom onset, marked systemic inflammatory response, absence of pericardial calcification on CT, and presence of an active exudative component, the multidisciplinary heart team favoured a transient inflammatory constrictive phenotype over chronic fibrotic disease. Treatment was initiated with standard weight-based quadruple antituberculous therapy (rifampicin 600 mg/day, isoniazid 300 mg/day, pyrazinamide 1600 mg/day, and ethambutol 1200 mg/day), oral prednisone (1 mg/kg/day, 60 mg daily), and intravenous furosemide.

**Figure 3:**
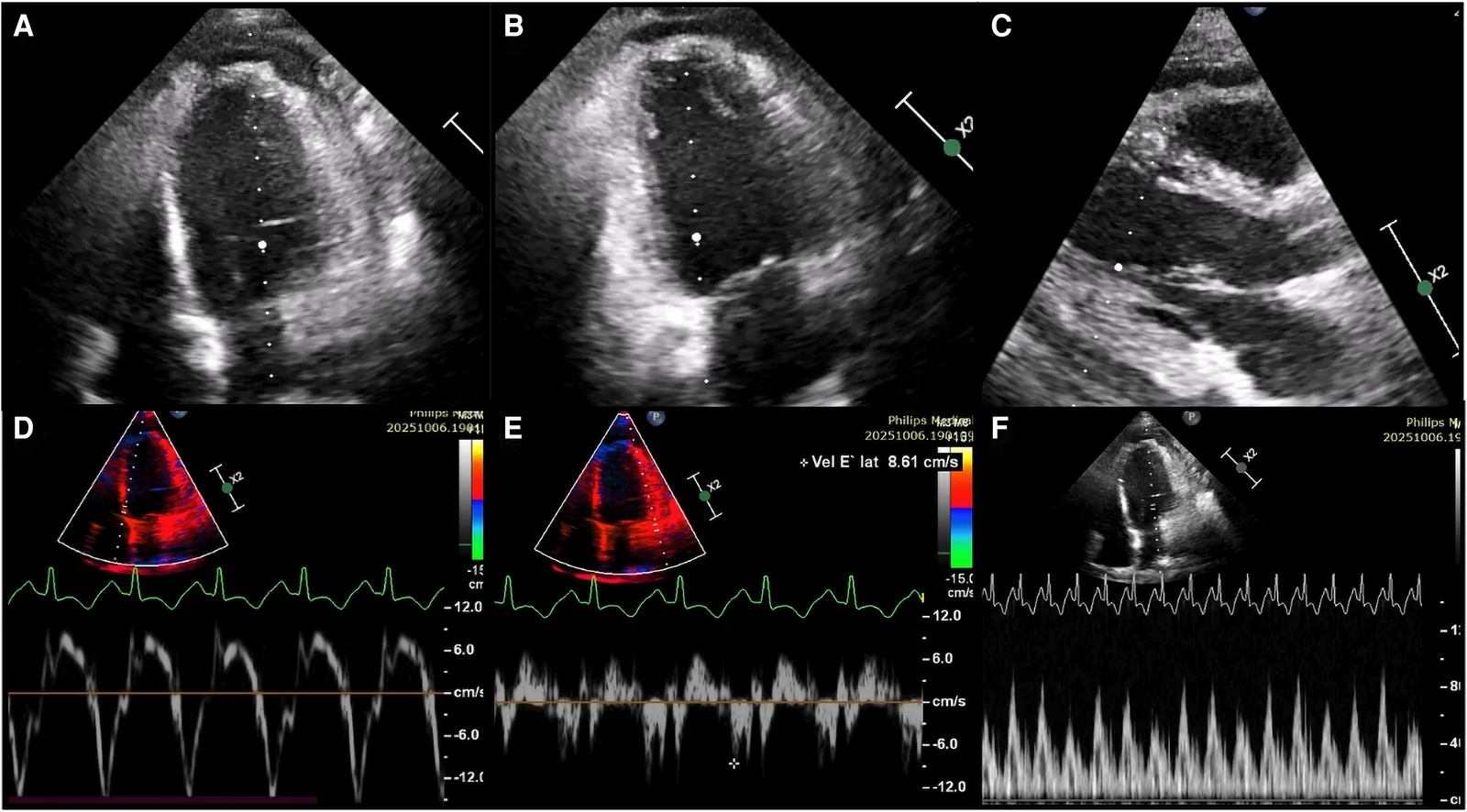
Six-panel echocardiographic study. (*Panels A*, *B*, and *C*) show a thickened, hyperechogenic pericardial lining and loculated effusion. (*Panels D* and *E*) show tissue Doppler imaging of the mitral annulus demonstrating *annulus reversus*, with a higher septal e′ velocity (17 cm/s) compared to the lateral e′ velocity (10 cm/s). (*Panel F*) shows pulsed-wave Doppler of mitral inflow with significant respiratory variation (29%), confirming constrictive physiology.

During intensive care unit monitoring, she required careful fluid titration to balance respiratory distress and symptomatic hypovolemia. After 2 weeks, she showed clinical improvement and was weaned from oxygen. A transient febrile decline due to an extended-spectrum beta-lactamase *Escherichia coli* urinary tract infection was successfully treated with ertapenem. Following a 1-month hospitalization, she achieved clinical remission. Repeat echocardiography demonstrated resolved *annulus reversus* and normalized flow velocities, with residual septal bounce. She was discharged with a stepwise 12-week prednisone taper (tapered by 10 mg every 2 weeks until reaching 20 mg, then by 5 mg every 2 weeks until discontinuation) and completed a 6-month TB regimen (2 months of rifampicin, isoniazid, pyrazinamide, and ethambutol, followed by 4 months of rifampicin and isoniazid), remaining asymptomatic with total echocardiographic resolution. The full-term neonate was healthy at birth, showed no evidence of congenital infection, and received appropriate paediatric care and follow-up. The patient was lost to follow-up after treatment completion.

## Discussion

Active TB affects approximately 216 500 pregnant women annually, contributing to 6–15% of maternal deaths in endemic areas.^4^ The incidence rate ratio for active disease significantly rises from 1.4 during pregnancy to 1.9 in the puerperium.^5^ This heightened puerperal susceptibility is probably driven by immune reconstitution following gestational T-cell suppression and Th2-dominance, a rebound that frequently unmasks latent TB and amplifies tissue inflammation. In our patient, this postpartum immune restoration provides a plausible biological mechanism for the fulminant presentation and acute onset of transient constrictive physiology.^4^

Pericardial involvement occurs in 1–8% of active TB cases and represents a significant diagnostic challenge during pregnancy.^1^ Transient constrictive pericarditis is a reversible inflammatory stage where constriction resolves with medical therapy before permanent fibrosis or calcification.^2^ This fibrinopurulent or ‘effusive-constrictive’ phase involves impaired compliance driven by acute inflammation and oedema rather than cicatricial remodelling; consequently, aggressive pharmacological treatment can restore pericardial elasticity.^6^ Echocardiography remains the first-line imaging modality for characterizing constrictive haemodynamics, demonstrating enhanced ventricular interdependence (*septal bounce*), respirophasic mitral inflow velocity variation (>25%), and preserved or augmented medial mitral annular velocity with *annulus reversus* (*e*′ medial > *e*′ lateral). As highlighted in current guidelines, multimodality imaging (echocardiography and CT/CMR) is essential to identify the transient, non-calcified inflammatory phenotype and differentiate it from irreversible chronic fibrocalcific constriction. Furthermore, while previous guidelines on cardiovascular diseases during pregnancy omitted specific frameworks for pericardial diseases, the updated 2025 ESC guidelines specifically address inflammatory syndromes in pregnancy and lactation, emphasizing tailored anti-inflammatory strategies during the puerperium.^2^

In high-burden settings, clinical prediction tools and biochemical biomarkers facilitate early TBP diagnosis. The Tygerberg Diagnostic Index Score (threshold ≥6) provides 86% sensitivity and 84% specificity for identifying tuberculous aetiology.^7^ Complementarily, ADA levels >40–50 U/l in serous fluids—as observed in our patient—yield high diagnostic accuracy (sensitivity 87%, specificity 89%), enabling prompt intervention when conventional microbiological yields are low.^2,7^ Although immune-based tests such as the tuberculin skin test and blood interferon-gamma release assays (IGRAs) are widely utilized, their clinical utility is markedly diminished in miliary TB due to high false-negative rates (up to 30–40%) secondary to transient systemic T-cell anergy. Moreover, in endemic populations, IGRAs cannot differentiate active disease from latent infection. Hence, therapeutic decisions rely on high pre-test clinical likelihood, pathognomonic imaging, elevated effusion ADA, and/or confirmed TB in another site, rather than awaiting delayed cultures.^1^

Standard management relies on a 6-month antituberculous regimen (rifampin, isoniazid, pyrazinamide, and ethambutol). Consistent with current guideline recommendations, adjunctive systemic corticosteroids serve as the primary anti-inflammatory strategy in HIV-negative tuberculous pericarditis, dampening the host immune response and mitigating the development of chronic pericardial constriction.^2^ The Investigation of the Management of Pericarditis (IMPI) trial demonstrated that steroids reduce constrictive pericarditis incidence and hospitalization frequency by promoting exudate resorption during the fibrinopurulent phase.^8^ For the transient constrictive phenotype, a ‘medical-first’ strategy is preferred, as early anti-inflammatory therapy reverses mechanical resistance from pericardial oedema and fibrin before irreversible fibrosis ensues. Pericardiectomy is reserved for chronic constriction with persistent NYHA III–IV symptoms, dense calcification, or failure after a 4-to-8-week optimized medical trial.^2,9^ Given the 6–12% surgical mortality in active TB and the physiological stress of the puerperium, this case underscores the importance of identifying the reversible window to avoid high-risk surgery.^9^

Peripartum tuberculous pericarditis is a rare, life-threatening condition frequently misdiagnosed due to symptom overlap with common gestational pathologies. The peripartum period—conventionally defined as the clinical window spanning from the late third trimester of pregnancy (≥28–36 weeks of gestation) through the first months postpartum—represents a phase of profound haemodynamic and immunological flux. In our patient, the clinical course accurately reflected this timeline. Although early pregnancy cases often lead to miliary TB or stillbirth, cardiac manifestations typically present during the third trimester or puerperium, coinciding with peak maternal haemodynamic stress.^10,11^ In our patient, the abrupt haemodynamic shifts following delivery likely unmasked severe diastolic restriction. Concurrently, the immediate postpartum reversal of gestational immunosuppression could have provoked an immune rebound akin to an immune reconstitution inflammatory syndrome, driving the sudden inflammatory surge and clinical deterioration observed in our case. This clinical course aligns with previously reported manifestations of fulminant postpartum tuberculous myopericarditis.^12^

The transient constrictive phenotype remains significantly underreported in obstetric literature compared with irreversible chronic forms. While established constriction typically necessitates high-risk pericardiectomy, the haemodynamic adaptations of labour can precipitate cardiovascular collapse in patients with a fixed stroke volume. Consequently, a ‘medical-first’ approach during the reversible phase is paramount to avoid the significant morbidity and mortality associated with surgical intervention in the peripartum period.^11,13^

Serial echocardiographic monitoring in our case documented the transition from constriction to near-complete functional resolution, a trajectory consistent with the longitudinal findings of Kim *et al*. (2020).^14^ Their research highlights that tuberculous pericarditis, particularly in its early fibrinopurulent phase, represents a dynamic inflammatory process with significant potential for reverse remodelling under optimized therapy, rather than an invariably terminal stage. The prompt normalization of *annulus reversus* and Doppler filling dynamics despite a persistent septal bounce aligns with favourable trajectories described by Strang *et al.* and Kim *et al*. This asynchronous recovery carries no negative prognostic significance in asymptomatic patients with normalized filling pressures. Rather, it highlights that timely antituberculous therapy and corticosteroids can successfully reverse transient inflammatory constriction and prevent disease progression.^14,15^

Limitations of this report must be acknowledged. As an isolated case presentation, these findings demonstrate the biological plausibility and clinical feasibility of successful medical management but cannot establish universal treatment algorithms or replace randomized trial evidence. The medical-first strategy requires strict patient selection and mandates serial monitoring to detect early signs of clinical deterioration that would warrant urgent pericardiectomy.

## Conclusion

Transient tuberculous constrictive pericarditis in the peripartum period represents a diagnostic challenge, as physiological adaptations mask evolving inflammation. This case demonstrates that tuberculous constriction often represents a reversible inflammatory phase responsive to medical management rather than an irreversible terminal stage. Timely pharmacological intervention with antituberculous agents and corticosteroids can achieve complete haemodynamic resolution, effectively obviating the need for high-risk surgical pericardiectomy.

## Lead author biography


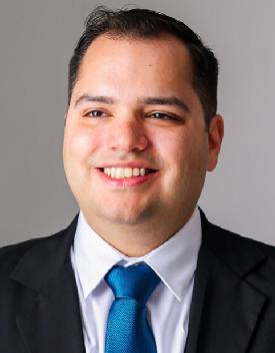
Jose Gabriel Solis is a clinical cardiologist and researcher at the Department of Cardiology, Hospital General de Matamoros, Mexico. He trained in Internal Medicine at the National Autonomous University of Mexico (UNAM) and subspecialized in Cardiology at the Autonomous University of Nuevo León (UANL). A member of the National System of Researchers (SNI), his academic work centers on echocardiography, cardiovascular risk, heart failure, driven by a deep passion for medical education.

## Supplementary Material

ytag794_Supplementary_Data

## Data Availability

All data supporting the findings of this study are included within the article and its accompanying figures. Further de-identified clinical details are available from the corresponding author upon reasonable request.
